# Chronic disease and malnutrition biomarkers among unemployed immigrants and Canadian born adults

**DOI:** 10.1186/s13690-019-0367-8

**Published:** 2019-09-18

**Authors:** Drissa Sia, Malgorzata Miszkurka, Malek Batal, Hélène Delisle, Maria Victoria Zunzunegui

**Affiliations:** 1 Département des sciences infirmières, Campus de Saint-Jérôme, Université du Québec en Outaouais, 5, rue Saint-Joseph, bureau J-3226, Saint Jérôme, Québec J7Z 0B7 Canada; 2 Département de médecine sociale et préventive, Université de Montréal, Montréal, Québec Canada; 3 Département de nutrition, Faculté de Médecine, Université de Montréal, Montréal, Québec Canada; 4 Département de médecine sociale et préventive, Université de Montréal, Montréal, Québec Canada; 5 École de santé publique, Université de Montréal, Montréal, Québec Canada

**Keywords:** Unemployment, Biomarkers, Immigrants, Gender, Canadian health measurement survey, Canada

## Abstract

**Background:**

Immigration status and unemployment may intersect on the health outcomes of men and women. This study aimed to identify intersections between unemployment and immigration in inflammatory, metabolic and nutritional blood markers and assess gender differences.

**Methods:**

We used Canadian Health Measures Survey data on 2493 participants aged 18 to 65. Outcomes were chronic inflammation (high-sensitivity C-reactive protein (hsCRP) and fibrinogen), nutritional (albumin and hemoglobin), and metabolic blood markers (glycosylated hemoglobin, blood glucose, total and high density lipoprotein (HDL) cholesterol). Multivariate linear regressions were used to assess the associations between each biomarker, unemployment and immigrant status, controlling for age, education, province, smoking, physical inactivity and body mass index and testing for multiplicative interactions between unemployment, immigrant status and gender.

**Results:**

Unemployment was associated with higher inflammation (hsCRP and fibrinogen) in Canadian born men; Canadian born employed women showed higher hsCRP values compared with corresponding employed men. Unemployed immigrant women presented the highest values of hsCRP while employed immigrant women had the lowest hsCRP. Unemployment was associated with higher glucose; immigrant status was associated with higher glucose and glycosylated hemoglobin. Unemployed immigrants had significantly lower levels of hemoglobin and albumin than employed immigrants, and Canadian-born citizens regardless of their employment status. Some of these associations were attenuated after adjustment by body mass index, physical inactivity and smoking.

**Conclusion:**

Blood biomarkers unveil intersections among unemployment, immigration and gender. This study provides evidence on biological pathways of unemployment on the likelihood of common chronic diseases, inflammation and potential malnutrition with some increased vulnerabilities in unemployed immigrants, and particularly in unemployed immigrant women.

## Background

Employment is a social determinant of health [1]. The effect of unemployment on health extends over long periods [2, 3]. Smoking, being overweight [4], and health conditions such as hypertension and coronary heart disease, mood disorders such as anxiety and depression, and suicide [5, 6] are among known behavioural and health adverse outcomes associated with unemployment. Most research has analyzed the links between unemployment and self-reported indicators of health [7] yet few studies have focused on the relationship between unemployment and health biomarkers. Most studies were limited to inflammatory markers showing that unemployment contributed to elevated inflammation markers such as C–reactive protein (CRP), fibrinogen and interleukin-6 [8, 9].

Being an immigrant may lead to an increased risk of chronic diseases [10–13]. In Canada, it has been reported that the health of immigrants upon their arrival is better than that of Canadian-born individuals of similar age and gender, and deteriorates over time to match that of the Canadian-born [14]. This phenomenon, known as the ‘healthy immigrant effect’, has been repeatedly reported in Canadian literature [15, 16]. What is less clear is what happens to some women and men as they gradually settle in Canada and which risk conditions are leading to this deterioration in health. Some have argued that new social environments (i.e. financial, education systems), an unstable income or economy and lack of social support contribute to a loss of physical and mental health advantages among immigrants [17–19]. A larger proportion of immigrants compared to Canadian-born individuals is unemployed and have lower wages and lower quality of employment. In 2012, the duration of unemployment among immigrants in Quebec was 26.4% longer than that of natives; a larger gap than in British Columbia (15.0%) and Ontario (10.4%) [20–22]. Low socioeconomic status of immigrants, low job satisfaction and unemployment have all been associated with the deterioration of health in the immigrant population [22, 23].

Social determinants of health and socioeconomic factors have been extensively analyzed, including the influential 2008 World Health Organization (WHO) report [24]. Most of this body of research has focused on immigration as a determinant of population health [25, 26] or on unemployment and health [27–29] and a rather large literature body has been related to gender-related risk factors for health [30–32] since gender is related to social context. Gender is not independent of other social determinants of health such as unemployment or immigrants status. We hypothesize that the intersection of unemployment and immigration status interact with gender in producing health outcomes since the social structures that characterize relationships between men and women influence the joint effects of immigration and employment on health. Moreover, unemployment has a stronger effect on men’s health compared to women’s [33, 34]. Gender differences in the impact of unemployment on health have been related to gender division of family responsibilities [33], which are more common in immigrant populations [35]. Finally, some evidence has shown gender differences in the association between stress and inflammation [36], and that immigration has different impacts on the health of men and women [37].

Research that focuses on immigration and unemployment separately and does not take gender into account is insufficient because these circumstances operate together and may have differential impact on the health outcomes of men and women. Few research efforts have simultaneously examined the social intersecting conditions of being unemployed and being an immigrant, and their combined effect on health [6] in men and in women. The current paper encompasses these three social determinants of health and examines how they interact to increase vulnerability to chronic diseases as assessed by blood biomarkers of nutritional, metabolic and inflammatory markers.

The objectives of this work were two fold 1) to assess whether unemployment has a stronger association with inflammatory, metabolic and nutritional markers in immigrants than in Canadian-born individuals; and 2) to examine how these biomarkers are related to employment and immigrant status in men and women by taking into account behavioral and socioeconomic factors [38].

## Methods

### Data

We used data from cycle 1 of the Canadian Health Measures Survey (CHMS) collected between 2007 and 2009 [39]. The CHMS was designed to cover a nationally representative sample of the Canadian population. Approximately 96% of Canadians aged 6 to 79 years and living in private households were included in its sampling frame.

Data collection was performed in two steps: first a Statistics Canada interviewer visited the respondents’ home and administered the health questionnaire; at the end of the visit, the interviewer informed the respondent that he/she had been randomly assigned to a visit at the mobile examination centre (MEC) for measurement of fasting glucose, blood lipids and insulin levels, among other tests. To make sure that respondents were in the fasting state, a morning appointment required that respondents fast overnight, and shorter eating restrictions were imposed on those with afternoon appointments [40]. Approximately 85% of respondents who completed the household interview agreed to go to the MEC [41]. Upon agreement, the blood (80 ml) was collected by a phlebotomist using a standardized venipuncture technique. The complete blood count was analyzed for all respondents from whom a sample was collected. This allowed measuring biomarkers such as glycosylated hemoglobin and hemoglobin.

Blood aliquots were packed and sent to Health Canada in Ottawa for chronic disease, general health and nutritional lab results [41]. A total of 15 collection sites in five Canadian provinces were selected. Based on 2006 census data, dwellings with known household composition were randomly selected. Survey participants were then selected among a list of current household members of each selected dwelling [42]. Plasma was used to assess high-sensitivity C-reactive protein (hsCRP), fibrinogen, blood glucose, total cholesterol and high density lipoprotein (HDL). The measurements of all biomarkers are those chosen by Statistics Canada and validated for the population survey [40].

As we are focusing on the relationship between unemployment and immigration with health biomarkers, we considered respondents aged 18 to 65 years with a total of 2493 respondents.

### Measures

In this study, we chose to examine the continuous distributions of eight blood biomarkers of common chronic conditions as outcomes and to focus on the public health importance of shifts of these population distributions. Similar to what Rose stated for the importance of shifting the cholesterol distribution to prevent deaths from coronary heart disease, we propose that a small decrease in the population average of these eight biomarkers’ concentrations could achieve much more than a screening policy and treating those with pathologically high levels of these biomarkers [43]. Based on the literature, we grouped the outcome variables into three categories: inflammatory biomarkers [44, 45]; metabolic biomarkers [46, 47] and nutritional biomarkers [48] although there is some overlap. These dependent variables are summarized in Table 1.

**Table 1:** Summary of outcomes: measurement and health risks

Biomarkers	Unit of measure	Kit used to measure	Normal value		Health risk
			Women	Men	
*Inflammatory markers*					
C-reactive protein	mg/L	hsCRP Reagent from VITROS Chemistry Products	< 5 mg /L		< 1.0 mg/L: lowest risk 1.0–3.0 mg/L: average risk ≥ 3 mg/L: highest risk
Fibrinogen	g/L	Sysmex CA-500 SERIES analyzer	2–4 g/L		Elevated levels increases the risk of cardiovascular disease Low level can be related to malnutrition or liver disease
*Metabolic markers*					
Blood glucose	mmol/L	VITROS Chemistry Systems	3.5–6 mmol/L		5,6–6 mmol/L: abnormal ≥ 7 mmol/L: provisional diagnosis of diabetes
Glycosylated hemoglobin	percentage of hemoglobin A1	VITROS Chemistry Products	4.5–6%		6- -7%: diabetes > 8%: poorly controlled diabetes
Total Cholesterol	mmol/L	VITROS Chemistry Products	< 5.18 mmol/L		5.18–6.19 mmol/L: upper limit > 6.20 mmol/L: high value
HDL Cholesterol	mmol/L	VITROS dHDL slide	> 1.30	> 1.03	Low level increases cardiovascular risk
*Nutritional markers*					
Albumin	g/L	VITROS ALB Slides and the VITROS Chemistry Products Calibrator Kit 4 on VITROS Chemistry Systems	40–50 g/L		Lower level can be a sign of malnutrition, or severe inflammation
Hemoglobin	g/L	–	120 – 160 g/L	130–180 g/L	Lower level is a sign of anemia

### Main independent variables

A person was considered to be unemployed if he/she had not worked throughout the preceding year but was available for work; or had worked in a family business without pay. However, people who were in school during the same period were excluded from this group. Immigrant status was assigned to each person born outside of Canada based on their reported place of birth.

### Adjustment for covariates

The following variables were selected according to the literature as potential confounders of the relationship between unemployment and health biomarkers are shown in Table 2. They include socio-demographic variables (age, education, and place of residence of residence), body mass index (BMI), smoking, physical activity, and place of residence.

**Table 2:** Unemployment in the Canadian Health Measures Survey (*N* = 2493)

	Total	Unemployed	*P*-value
Age (years)			0.018
18–29	586	117 (19.97)	
30–44	956	180 (18.83)	
45–55	627	123 (19.62)	
56–65	324	87 (26.85)	
Gender			0.165
Men	1234	237 (19.21)	
Women	1259	270 (21.45)	
Immigrant			0.013
No	2000	387 (19.35)	
Yes	492	120 (24.39)	
Education			< 0.001
< High school	127	48 (37.80)	
High school	1028	220 (21.40)	
Some college	628	115 (18.31)	
University	699	123 (17.60)	
Province			0.624
Ontario	919	191 (20.78)	
Quebec	664	139 (20.93)	
Alberta	363	66 (18.18)	
British Columbia	354	77 (21.75)	
New Brunswick	193	34 (17.62)	
BMI			
< 30 Kg/m^2^	1896	366 (19.30)	0.014
30 kg/m^2^ and over	561	135 (20.06)	
Physical activity			
Very active	503	103 (20.48)	0.915
Active	631	132 (20.92)	
Inactive	1283	226 (20.11)	
Smoking			
No	1882	343 (18.23)	< 0.001
Yes	575	158 (27.48)	

### Statistical analysis

Descriptive analyses were done in two steps. First, the Chi^2^ test was used to compare the proportion of unemployment by categories of each potential confounder of the association between unemployment and poor blood biomarkers. Second, hsCRP, blood glucose and glycosylated hemoglobin were transformed to the logarithm scale to avoid skewness.

We used an analytical strategy taking into account intersections with unemployment, immigration and gender to quantify their joint influences on health. Since the distributions of these biomarkers or their log-transformed variables were unimodal, we fitted multiple linear regression equations to assess the relationship between each biomarker, unemployment, immigrant status and gender. To test for differences in their means by unemployment status, immigrant status and gender, we estimated the means for the reference group (Canadian-born employed men) as intercepts and the deviations from these means as coefficients of the indicators of unemployment, immigrant status and gender and their interactions.

Multiple linear regressions were fitted in three steps: first, including indicators of unemployment, immigration and gender whose interactions constitute our main hypothesis; second, adding terms to the equations resulting of the first step to control for age, sex and education and third, further adding terms to control for body mass index, smoking and physical activity. Thus, “model a” included unemployment, immigrant status, gender and the multiplicative interaction terms for the first order interactions between unemployment and immigration, unemployment and gender, immigration and gender; and the second order interaction terms between unemployment, immigration and gender; each interaction was included if significant at a *p*-value < 0.05; (2) in “model b”, age, education and province of residence, were added to model “a”; and (3) in “model c” BMI and behavioural variables were added to “model b”.

Regression analyses were based on unweighted data because sampling was a function of independent variables included in the model, producing unbiased coefficients and standard errors [49]. All analyses were done using Stata 12 [50].

## Results

### Sample characteristics

The proportion of unemployed adults was higher among immigrants (24.4%) and respondents over 55 years (26.9%) (Table 1). The unemployment was similar among immigrant and Canadian born men whereas marked difference were reported between immigrant and Canadian born women (19.8% of 1016 Canadian born versus 30% of 213 immigrants were unemployed (*p* = 0.001)). Unemployment was more frequent among those older than 55, smokers and obese respondents, and decreased with increasing levels of education (Table 2).

### Blood biomarker associations with unemployment and immigrant status: multivariate regression analyses

To ease interpretation of coefficients in models including tests of first order (unemployment*immigration, unemployment*gender, immigration *gender) and second order multiplicative interactions (unemployment*immigration*gender), we present in Table 3 the estimated deviations from the biomarkers mean of the Canadian born employed men (the reference group) resulting from the fit of a multivariate regression model including the three main exposure variables under study: unemployment, immigrant status and gender. To calculate the means presented in Table 3 for hsCRP, blood glucose and glycosylated hemoglobin whose values had been log transformed, we converted the mean differences back to the natural scale. Converting the differences back to the natural scale produces the real biological values, which allow for a better interpretation of their biological meaning. For example, the calculations for glycosylated hemoglobin were done as follows: a) the intercept of log transformed glycosylated hemoglobin regression equation was (− 2.9), indicating that the mean value for Canadian employed men was exp.(− 2.9) = 5.5; b) The coefficient of unemployed immigrant men and women in that equation was 0.26, then the mean for those groups would be exp.(− 2.9 + 0.26) = 7.13 and the difference between groups would be 7.13–5.5 = 1.63, as shown in Table 3.

**Table 3:** Estimated deviations from the biomarkers mean of employed Canadian men: Mean deviations of the unemployed Canadian born and employed and unemployed immigrant men and women

	Canadian born		Immigrant	
	Men	Women	Men	Women
hsC Reactive Protein (mg/L)				
Employed	0	0.25*	0.0	−0.32*
Unemployed	0.25*	0.13	−0.26	1.00**
Fibrinogen (g/L)				
Employed	0	0	0	0
Unemployed	0.1**	0.1**	0.07	0.07
Glycosylated hemoglobin (%)				
Employed	0	0	0.89*	0.89*
Unemployed	0.64	0.64	1.63*	1.63*
Glucose (mmol/L)				
Employed	0	0	1.023**	1.023**
Unemployed	1.022**	1.022**	1.045**	1.045**
Hemoglobin (g/L)				
Employed	0	0	0.09	0.09
Unemployed	0.3	0.3	−5.89***	−5.89***
Albumin (g/L)				
Employed	0	0	0.24	0.24
Unemployed	−0.12	−0.12	−0.73*	−0.73*

*: < 0.05; ** <  0.01; ***: < 0.001

Mean values for Canadian born employed men: C - reactive protein = 1.07(mg/L); Fibrinogen = 2.87(g/L); Glycosylated hemoglobin =5.5(%); Glucose = 4.842 mmol/L; Hemoglobin 142.82 (g/L); Albumin 46.57 (g/L) as estimated in model “a” of Table 3

The mean deviation of hsCRP for Canadian born unemployed men was 0.25 mg/L compared with Canadian born employed men; Canadian born employed women had a hsCRP mean 0.25 mg/L higher than that of Canadian born employed men. Unemployment had opposite effects in hsCRP among immigrant women: hsCRP was highest among unemployed immigrant women (+ 1.0 mg/L) and lowest among those employed (− 0.32 mg/L), compared with the reference group, Canadian born employed men. For fibrinogen, Canadian born unemployed men had higher values than corresponding employed men, with a difference of 0.1 g/L.

Concerning glucose metabolism, fasting glucose was significantly higher (+ 1.023 mmol/L) in employed immigrant men and women compared with employed Canadian men. Glucose was also significantly higher in unemployed men and women (both Canadian born and immigrant) compared with those employed. Thus, unemployed immigrants’ glycemia was 1.045 mmol/L higher and that of unemployed Canadians 1.022 mmol/L higher than the mean value (4.842 mmol/L) for Canadian-born employed men. Similar associations were observed for glycosylated hemoglobin in employed and unemployed immigrants, who had significantly elevated values compared with Canadian-born men and women. The difference in population means reaches 1.63% in the case of unemployed immigrants, which represents an increment of 30% over the reference Canadian employed population (1.63/5.5%).

Lastly, hemoglobin and albumin were significantly lower by 5.89 g/L and 0.73 g/L, respectively in unemployed immigrant men and women compared with the Canadian-born groups. No differences between the comparison groups were detected in lipid metabolism, as assessed by total cholesterol and HDL cholesterol (data not shown).

Table 4 shows adjusted beta coefficients in three series of models. Model “a” includes only employment, immigrant status and gender and it is the basis for the values shown in Table 3. Model b controls for age, education and province of residence and model c controls for BMI and smoking and physical activity. The interaction between unemployment, immigrant status and gender for hsCRP levels retained statistical significance in the fully adjusted “model c”, but the difference in hsCRP between unemployed and employed Canadian men was attenuated after adjustment by BMI, physical activity and smoking (model c).

**Table 4:** Biomarker associations with unemployment and immigration: Coefficients from multiple regression analyses in the Canadian Health Measures Survey

	*hsCRP* ^*a*^			*Fibrinogen*			*Glucose* ^*a*^		
	β (ES)			β (ES)			β (ES)		
	Model a (*n* = 2219)	Model b (*n* = 2219)	Model c (*n* = 2219)	Model a (*n* = 2374)	Model b (*n* = 2374)	Model c (*n* = 2374)	Model a (*n* = 2429)	Model b (*n* = 2429)	Model c (*n* = 2429)
Unemployed									
Yes vs. No	0.2084 (0.1008)*	0.2016 (0.0998)*	0.1033 (0.0939)	0.10 (0.03)**	0.07 (0.03)*	0.05 (0.03)	0.0217 (0.0081)**	0.0167 (0.0079)*	0.0141 (0.0078)
Immigrant									
Yes vs. No	−0.0358 (0.0924)	− 0.0014 (0.0930)	0.0134 (0.0874)	− 0.03 (0.03)	− 0.04 (0.03)	− 0.02 (0.03)	0.0226 (0.0083)**	0.0126 (0.0082)	0.0168 (0.0082)*
Unemployed and Immigrant									
Yes vs. No	−0.2877 (0.2109)	−0.3212 (0.2080)	− 0.2597 (0.1947)	–	–	–	–	–	–
Gender									
Woman vs. Man	0.2120 (0.0607)***	0.2346 (0.0601)***	0.2376 (0.0565)***	–	–	–	–	–	–
Woman and Unemployed									
Yes vs. No	−0.1329 (0.1388)	−0.1544 (0.1372)	− 0.0425 (0.1286)	–	–	–	–	–	–
Woman and Immigrant									
Yes vs. No	−0.3485 (0.1437)*	−0.3960 (0.1421)**	− 0.3646 (0.1331)**	–	–	–	–	–	–
Woman and Immigrant and Unemployed									
Yes vs. No	0.6580 (0.2939)*	0.6600 (0.2897)*	0.5625 (0.2714)**	–	–	–	–	–	–
	*Albumin*						*Hemoglobin* ^*a*^		
	β (ES)						β (ES)		
	Model a (*n* = 2400)	Model b (*n* = 2400)	Model c (*n* = 2400)				Model a (*n* = 2.401)	Model b (*n* = 2.401)	Model c (*n* = 2.401)
Unemployed									
Yes vs. No	−0.1215 (0.1786)	−0.0624 (0.1655)	0.0301 (0.1641)				0.2956 (0.7392)	0.4384 (0.5348)	0.1076 (0.5358)
Immigrant									
Yes vs. No	0.2381 (0.1832)	0.2622 (0.1736)	0.2234 (0.1716)				0.0912 (0.7583)	−1.4745 (0.5608)**	−1.4159 (0.5604)*
Unemployed immigrant									
Yes vs. No	−0.7303 (0.3797)*	−0.4155 (0.3505)	−0.4635 (0.3452)				−5.8976 (1.5594)***	−3.4097 (1.1235)**	−3.1404 (1.1184)**
	*Glycosylated hemoglobin* ^*a*^			*Total Cholesterol*			*HDL Cholesterol*		
	β (SE)			β (SE)			Β (SE)		
	Model a (*n* = 2368)	Model b (*n* = 2368)	Model c (*n* = 2368)	Model a (*n* = 2405)	Model b (*n* = 2405)	Model c (*n* = 2405)	Model a (*n* = 2405)	Model b (*n* = 2405)	Model c (*n* = 2405)
Unemployed									
Yes vs. No	0.109 (0.071)	0.066 (0.069)	0.059 (0.069)	0.0655 (0.0515)	0.06 (0.0482)	0.0445 (0.0483)	0.0023 (0.0186)	−0.0089 (0.0174)	−0.004 (0.0169)
Immigrant									
Yes vs. No	0.147 (0.073)*	0.159 (0.073)*	0.163 (0.073)*	0.0885 (0.0524)	0.0132 (0.0504)	0.0253 (0.0506)	−0.0215 (0.0189)	−0.0192 (0.0182)	− 0.0194 (0.0177)

*: < 0.05; ** <  0.01; ***: < 0.001^a^ The natural logarithm was used to correct the distribution; β: beta; SE: standard error

Unemployed immigrants had lower level of hemoglobin in the fully adjusted model while the lower levels of albumin in unemployed immigrants lost significance after adjustment by sociodemographic variables.

The mean values of glucose and glycosylated hemoglobin in immigrants compared with Canadian born men and women were still significantly higher in the fully adjusted model c. The significance of the association between glucose and unemployment was attenuated after adjustment by BMI, smoking and physical activity.

## Discussion

Summarizing our results, unemployed immigrant women and Canadian men showed the highest levels of inflammation. Unemployment and immigrant status intersected being associated with higher glucose and glycosylated hemoglobin. Unemployment and immigrant status intersected, and were associated with the lowest hemoglobin and albumin levels.

Our results on higher inflammation among Canadian born unemployed men are consistent with prior studies [8, 9]. Chronic stress can stimulate inflammatory reactions [51, 52]. Chronic inflammation has also been shown to lead to increased risk of chronic diseases and poor physical function [53, 54]. By increasing inflammation, unemployment may clinically contribute to faster health decline. These higher levels of inflammation in Canadian unemployed men were attenuated after adjustment by BMI, smoking and physical inactivity, consistent with the literature [55, 56]. In fact, for these health behaviours those who were born in Canada present two to three times higher rates compared to new or long term immigrants [57]. In our study, the difference in mean fibrinogen concentration between employed and unemployed Canadians was 0.1 g/L. This is similar to differences reported in the Caerphilly Prospective Heart Disease study, where fibrinogen concentration was lower by 0.14 g/L in the tertile of men who were most active in leisure activities. In this same study, fibrinogen concentration was between 0.1 g/L and 0.20 g/L lower in the employed than in the unemployed men whatever the level of leisure physical activity, and this difference was correlated with the prevalence of ischemic heart disease [58].

Among immigrant women, the estimated hsCRP difference between unemployed and employed women was 0.32 + 1.00 = 1.32 mg/l; which may be clinically significant since a difference of this magnitude has been related to lower physical function in old age [59]. This association between unemployment and inflammation in immigrant women has not been previously reported but may be related to the triple vulnerability of being unemployed, immigrant and woman. Associations between being employed and inflammation were positive among Canadian born women, an unexpected result that could be explained if Canadian women had higher level of inflammation related to a cumulative burden of work and family responsibilities.

Unemployed immigrants were at higher risk of diabetes (higher glucose and higher glycated hemoglobin), which could be linked to two immigration-related changes in their lives: first, their original diet shifts toward a diet that is richer in sugar, saturated fat and processed foods, low in fruit, vegetables and fiber [60]; and second, their physically active life becomes a sedentary life [61]. These dietary and behavioral transitions increase the risk of chronic conditions such as obesity, diabetes and cardiovascular disease [62, 63]. Moreover, their unemployment status may further reduce their chances to afford or access the quantity and quality of foods leading to poor diet as well as to sports and active leisure. Glycosylated hemoglobin differences associated with higher cardiovascular risk are around 0.50% and the difference between unemployed immigrants and employed Canadian-born subjects in this study is three times as high (around 1.63%). Therefore, such a difference between population means it may have clinical significance [64, 65]. Indeed, it has been shown that small shifts in population mean distribution, for instance for high blood pressure, made a clinically significant difference in the numbers at risk [66].

Results on lower hemoglobin and albumin may point to a higher risk of anemia among the unemployed immigrant population. Poverty among immigrants in Canada is higher than among Canadian-born citizens [67] and immigrants generally have lower social support [68], they live in more economically disadvantaged neighbourhoods [69] and may lack food security [70, 71]. In this scenario, lower hemoglobin values could be explained by the lack of money to buy adequate food.

Our approach aimed at examining the shift in the biomarker distributions in whole population groups, that is, a shift detected by a deviation of their means, using the strategy of preventive medicine proposed by Rose G [72] and expanded to vulnerable populations by Frohlich and Potvin [73]. The answer to our question has to do with the determinants of the population means, for what distinguishes the comparison groups “has nothing to do with the characteristics of the individuals, it is rather a mass influence acting on the population as a whole” [72].

There were several limitations to this study. First, if unemployed individuals were underrepresented in CHMS, the strength of the reported associations may be underestimated. Since only 12 subjects were unable to answer the survey in English or French, we would expect that immigrants who go for blood tests would be more likely to be fluent in English or French, and therefore more integrated in the labor market. A recent comparison between the self-rated health and health behaviors of the CHMS and the 2011 Canadian Community Health Survey show that both surveys over-represent the more privileged immigrants, since immigrant participation in these surveys is low [74]. Second, CHMS does not distinguish between types of immigrants. Our study definition of immigrants includes refugees, who have worse health than other types of immigrants [75]. Refugees represented 16.4% of all immigrants admitted to Canada between 1991 and 2000. Grouping these populations together allows for estimating an overall immigrant effect, but it may mask specific effects in subgroups according to ethnicity, time since immigration and age at immigration [12, 13, 76, 77]. No dietary data was analyzed for the purpose of this study, as nutritional data was unavailable in CHMS. The significant relationships between unemployment, immigration and blood biomarkers may not be causal due to potential reverse causality in a sense that some levels of biomarkers (good or bad health) could determine participant’s employment and/or immigrant status. Finally, as CHMS is a cross-sectional survey, we used one fasting blood glucose measurement to assess risk of diabetes and doing so, could introduce a measurement bias in the results. However, others authors also used a single determination of glucose to assess diabetes using data from CHMS [78] or data from The National Health and Nutrition Examination Survey (NHANES) [79].

## Conclusion

This is a preliminary study providing evidence for the biological pathways of unemployment on the likelihood of common chronic diseases, inflammation and potential malnutrition with some increased vulnerabilities in unemployed immigrants, and particularly in unemployed immigrant women. The effects of unemployment and\or immigration may generate a complex interplay between upstream living conditions, health behaviours and biological manifestations leading to stress, malnutrition and increased risk of chronic conditions. Health determinants such as access and affordability of good quality foods, in those immigrant populations who are unemployed needs to be further examined. Meanwhile, higher inflammation levels among those born in Canada and their underlying causes could also be examined more extensively. Ultimately, equal opportunities for employment for immigrant populations may reduce the observed differences in blood biomarkers according to employment, immigrant status and gender.

## Data Availability

The data that support the findings of this study are available under requested to Statistics Canada and the Quebec inter-University Centre for Social Statistics (QICSS).

## Abbreviations

BMI: Body mass index
CHMS: Canadian Health Measures Survey
HDL: High density lipoprotein
hsCRP: High-sensitivity C-reactive protein
MEC: Mobile examination centre
WHO: World Health Organization

## References

[1] Bartley M, Ferrie J (2010). Do we need to worry about the health effects of unemployment?. J Epidemiol Community Health.

[2] Hammarstrom A, Janlert U (2002). Early unemployment can contribute to adult health problems: results from a longitudinal study of school leavers. J Epidemiol Community Health.

[3] Helgesson M, Johansson B, Nordqvist T, Lundberg I, Vingard E (2013). Unemployment at a young age and later sickness absence, disability pension and death in native swedes and immigrants. Eur J Pub Health.

[4] Zagozdzon P, Parszuto J, Wrotkowska M, Dydjow-Bendek D (2014). Effect of unemployment on cardiovascular risk factors and mental health. Occup Med (Lond).

[5] Cable N, Sacker A, Bartley M (2008). The effect of employment on psychological health in mid-adulthood: findings from the 1970 British cohort study. J Epidemiol Community Health.

[6] Chen L, Li W, He J, Wu L, Yan Z, Tang W (2012). Mental health, duration of unemployment, and coping strategy: a cross-sectional study of unemployed migrant workers in eastern China during the economic crisis. BMC Public Health.

[7] Meneton P, Kesse-Guyot E, Mejean C, Fezeu L, Galan P, Hercberg S (2015). Unemployment is associated with high cardiovascular event rate and increased all-cause mortality in middle-aged socially privileged individuals. Int Arch Occup Environ Health.

[8] Hintikka J, Lehto SM, Niskanen L, Huotari A, Herzig KH, Koivumaa-Honkanen H (2009). Unemployment and ill health: a connection through inflammation?. BMC Public Health.

[9] Hughes A, McMunn A, Bartley M, Kumari M (2015). Elevated inflammatory biomarkers during unemployment: modification by age and country in the UK. J Epidemiol Community Health.

[10] Beiser M (2005). The health of immigrants and refugees in Canada. Can J Public Health.

[11] Bingham BA, Duong MT, Ricks M, Mabundo LS, Baker RL, Utumatwishima JN, et al. The association between stress measured by allostatic load score and physiologic dysregulation in African immigrants: the Africans in America study. Front Public Health. 2016;4:1-8. 10.3389/fpubh.2016.00265 PMC5122568 27933289

[12] Chiu M, Austin PC, Manuel DG, Tu JV (2010). Comparison of cardiovascular risk profiles among ethnic groups using population health surveys between 1996 and 2007. CMAJ.

[13] Creatore MI, Moineddin R, Booth G, Manuel DH, DesMeules M, McDermott S (2010). Age- and sex-related prevalence of diabetes mellitus among immigrants to Ontario, Canada. Cmaj.

[14] Fuller-Thomson E, Noack AM, George U (2011). Health decline among recent immigrants to Canada: findings from a nationally-representative longitudinal survey. Can J Public Health.

[15] McDonald JT, Kennedy S (2004). Insights into the ‘healthy immigrant effect’: health status and health service use of immigrants to Canada. Soc Sci Med.

[16] Saposnik G, Redelmeier DA, Lu H, Fuller-Thomson E, Lonn E, Ray JG (2010). Myocardial infarction associated with recency of immigration to Ontario. Qjm.

[17] Miszkurka M, Goulet L, Zunzunegui MV (2010). Contributions of immigration to depressive symptoms among pregnant women in Canada. Can J Public Health..

[18] Shishehgar S, Gholizadeh L, DiGiacomo M, Davidson PM (2015). The impact of migration on the health status of Iranians: an integrative literature review. BMC Int Health Hum Rights.

[19] Syed HR, Dalgard OS, Dalen I, Claussen B, Hussain A, Selmer R (2006). Psychosocial factors and distress: a comparison between ethnic Norwegians and ethnic Pakistanis in Oslo, Norway. BMC Public Health.

[20] Boudarbat B (2011). Les défis de l’intégration des immigrants dans le marché du travail au Québec : enseignements tirés d’une comparaison avec l’Ontario et la Colombie-Britannique, rapport de projet CIRANO, no 2011RP-07.

[21] Boudarbat B, Connolly M (2013). Évolution de l’accès a l’emploi et des conditions de travail des immigrants au Québec, en Ontario et en Colombie-Britannique entre 2006 et 2012. Série scientifique (CIRANO).

[22] Boudarbat B, Lemieux T (2014). Why are the relative wages of immigrants declining? A Distributional Approach. ILR Review.

[23] Booth GL, Creatore MI, Moineddin R, Gozdyra P, Weyman JT, Matheson FI (2013). Unwalkable neighborhoods, poverty, and the risk of diabetes among recent immigrants to Canada compared with long-term residents. Diabetes Care.

[24] World Health Organization. In: World Health Organization, editor. Closing the gap in a generation: health equity through action on the social determinants of health. Final Report of the Commission on Social Determinants of Health. Geneva; 2008. p. 256. https://www.who.int/social_determinants/thecommission/finalreport/en/.

[25] Ayón C (2018). Unpacking immigrant health: policy, stress, and demographics. Race Soc Probl.

[26] Merry L, Semenic S, Gyorkos TW, Fraser W, Small R, Gagnon AJ (2016). International migration as a determinant of emergency caesarean. Women Birth.

[27] Hollederer A (2011). Unemployment and health in the German population: results from a 2005 microcensus. J Public Health.

[28] Janlert U, Winefield AH, Hammarström A (2014). Length of unemployment and health-related outcomes: a life-course analysis. Eur J Pub Health.

[29] Premji S, Shakya Y (2017). Pathways between under/unemployment and health among racialized immigrant women in Toronto. Ethn Health.

[30] Barstad LH, Júlíusson PB, Johnson LK, Hertel JK, Lekhal S, Hjelmesæth J (2018). Gender-related differences in cardiometabolic risk factors and lifestyle behaviors in treatment-seeking adolescents with severe obesity. BMC Pediatr.

[31] Łoboz-Rudnicka M, Jaroch J, Kruszyńska E, Bociąga Z, Rzyczkowska B, Dudek K (2018). Gender-related differences in the progression of carotid stiffness with age and in the influence of risk factors on carotid stiffness. Clin Interv Aging.

[32] Pucci G, Alcidi R, Tap L, Battista F, Mattace-Raso F, Schillaci G (2017). Sex- and gender-related prevalence, cardiovascular risk and therapeutic approach in metabolic syndrome: a review of the literature. Pharmacol Res.

[33] Artazcoz L, Benach J, Borrell C, Cortès I (2004). Unemployment and mental health: understanding the interactions among gender, family roles, and social class. Am J Public Health.

[34] Kim TJ, von dem Knesebeck O (2015). Is an insecure job better for health than having no job at all? A systematic review of studies investigating the health-related risks of both job insecurity and unemployment. BMC Public Health.

[35] Ro A, Goldberg RE (2017). Post-migration employment changes and health: A dyadic spousal analysis. Soc Sci Med.

[36] Vassalle C, Simoncini T, Chedraui P, Pérez-López FR (2012). Why sex matters: the biological mechanisms of cardiovascular disease. Gynecol Endocrinol.

[37] Llácer A, Zunzunegui MV, del Amo J, Mazarrasa L, Bolůmar F (2007). The contribution of a gender perspective to the understanding of migrants’ health. J Epidemiol Community Health.

[38] Tremblay MS, Wolfson M, Connor GS (2007). Canadian health measures survey: rationale, background and overview. Health Rep.

[39] Bryan S, St-Denis M, Wojtas D (2007). Canadian health measures survey: clinic operations and logistics. Health Rep.

[40] Giroux S (2007). Canadian health measures survey: sampling strategy overview. Health Rep.

[41] Statistic Canada (2011). Canadian health measures survey (CHMS) data user guide: cycle 1.

[42] Tremblay MS, Connor GS (2007). Canadian health measures survey: brief overview. Can J Public Health..

[43] Rose G, Shipley M (1986). Plasma cholesterol concentration and death from coronary heart disease: 10 year results of the Whitehall study. Br Med J (Clin Res Ed).

[44] Adeli K, Higgins V, Nieuwesteeg M, Raizman JE, Chen Y, Wong SL (2015). Complex reference values for endocrine and special chemistry biomarkers across pediatric, adult, and geriatric ages: establishment of robust pediatric and adult reference intervals on the basis of the Canadian health measures survey. Clin Chem.

[45] Pearson TA, Mensah GA, Alexander RW, Anderson JL, Cannon RO, Criqui M (2003). Markers of inflammation and cardiovascular disease. Circulation.

[46] Da Costa LA, Arora P, García-Bailo B, Karmali M, El-Sohemy A, Badawi A (2012). The association between obesity, cardiometabolic disease biomarkers, and innate immunity-related inflammation in Canadian adults. Diabetes Metab Syndr Obes.

[47] Carson V, Wong SL, Winkler E, Healy GN, Colley RC, Tremblay MS (2014). Patterns of sedentary time and cardiometabolic risk among Canadian adults. Prev Med.

[48] Corona LP, de Oliveira Duarte YA, Lebrão ML (2018). Markers of nutritional status and mortality in older adults: the role of anemia and hypoalbuminemia. Geriatr Gerontol Int.

[49] Winship C, Radbill L (1994). Sampling weights and regression analysis. Sociol Methods Res.

[50] StataCorp (2009). Stata: Release 11. Statistical Software.

[51] McDade TW, Hawkley LC, Cacioppo JT (2006). Psychosocial and behavioral predictors of inflammation in middle-aged and older adults: the Chicago health, aging, and social relations study. Psychosom Med.

[52] Ranjit N, Diez-Roux AV, Shea S, Cushman M, Seeman T, Jackson SA (2007). Psychosocial factors and inflammation in the multi-ethnic study of atherosclerosis. Arch Intern Med.

[53] Brinkley TE, Leng X, Miller ME, Kitzman DW, Pahor M, Berry MJ (2009). Chronic inflammation is associated with low physical function in older adults across multiple comorbidities. J Gerontol A Biol Sci Med Sci.

[54] Danesh J, Wheeler JG, Hirschfield GM, Eda S, Eiriksdottir G, Rumley A (2004). C-reactive protein and other circulating markers of inflammation in the prediction of coronary heart disease. N Engl J Med.

[55] Aldaham S, Foote JA, Chow H-HS, Hakim IA (2015). Smoking status effect on inflammatory markers in a randomized trial of current and former heavy smokers. Int J Inflamm.

[56] Imhof A, Froehlich M, Brenner H, Boeing H, Pepys MB, Koenig W (2001). Effect of alcohol consumption on systemic markers of inflammation. Lancet.

[57] Public Health Agency of Canada, the Pan-Canadian Public Health Network, Statistics Canada, the Canadian Institute of Health Information (2017). Pan-Canadian health inequalities data tool. 2017 edition ed.

[58] Elwood PC, Yarnell JW, Pickering J, Fehily AM, O'Brien JR (1993). Exercise, fibrinogen, and other risk factors for ischaemic heart disease. Caerphilly prospective heart disease study. Br Heart J.

[59] Sousa AC, Zunzunegui MV, Li A, Phillips SP, Guralnik JM, Guerra RO (2016). Association between C-reactive protein and physical performance in older populations: results from the international mobility in aging study (IMIAS). Age Ageing.

[60] Delisle H (2010). Findings on dietary patterns in different groups of African origin undergoing nutrition transition. Appl Physiol Nutr Metab.

[61] Sodergren M, Sundquist K, Johansson SE, Sundquist J, Hagstromer M (2010). Associations between health-enhancing physical activity and country of birth among women. J Phys Act Health.

[62] Gushulak BD, Pottie K, Hatcher Roberts J, Torres S, DesMeules M (2011). Migration and health in Canada: health in the global village. Cmaj.

[63] Sundquist J, Winkleby M (2000). Country of birth, acculturation status and abdominal obesity in a national sample of Mexican-American women and men. Int J Epidemiol.

[64] Di Angelantonio E, Gao P, Khan H, Butterworth AS, Wormser D, Kaptoge S (2014). Glycated hemoglobin measurement and prediction of cardiovascular disease. Jama.

[65] Selvin E, Steffes MW, Zhu H, Matsushita K, Wagenknecht L, Pankow J (2010). Glycated hemoglobin, diabetes, and cardiovascular risk in nondiabetic adults. N Engl J Med.

[66] Laaser U, Breckenkamp J, Ullrich A, Hoffmann B (2001). Can a decline in the population means of cardiovascular risk factors reduce the number of people at risk?. J Epidemiol Community Health.

[67] Van Hulst A, Seguin L, Zunzunegui MV, Velez MP, Nikiema B (2011). The influence of poverty and social support on the perceived health of children born to minority migrant mothers. Ethn Health.

[68] Miszkurka M, Goulet L, Zunzunegui MV (2012). Antenatal depressive symptoms among Canadian-born and immigrant women in Quebec: differential exposure and vulnerability to contextual risk factors. Soc Psychiatry Psychiatr Epidemiol.

[69] Urquia ML, O'Campo PJ, Heaman MI (2012). Revisiting the immigrant paradox in reproductive health: the roles of duration of residence and ethnicity. Soc Sci Med.

[70] Sanou D, O'Reilly E, Ngnie-Teta I, Batal M, Mondain N, Andrew C (2014). Acculturation and nutritional health of immigrants in Canada: a scoping review. J Immigr Minor Health.

[71] Vahabi M, Damba C, Rocha C, Montoya EC (2011). Food insecurity among Latin American recent immigrants in Toronto. J Immigr Minor Health.

[72] Geoffrey R (2001). Sick individuals and sick populations. Bull World Health Organ.

[73] Frohlich KL, Potvin L (2008). Transcending the known in public health practice: the inequality paradox: the population approach and vulnerable populations. Am J Public Health.

[74] Ng E (2015). Canadian health measures survey: a tool for immigrant health research?. Health Rep.

[75] Okrainec K, Bell CM, Hollands S, Booth GL (2015). Risk of cardiovascular events and mortality among a population-based cohort of immigrants and long-term residents with diabetes: are all immigrants healthier and if so, for how long?. Am Heart J.

[76] Chiu M, Austin PC, Manuel DG, Tu JV (2012). Cardiovascular risk factor profiles of recent immigrants vs long-term residents of Ontario: a multi-ethnic study. Can J Cardiol.

[77] Chiu M, Maclagan LC, Tu JV, Shah BR (2015). Temporal trends in cardiovascular disease risk factors among white, south asian, chinese and black groups in ontario, canada, 2001 to 2012: a population-based study. BMJ Open.

[78] Rosella LC, Lebenbaum M, Fitzpatrick T, Zuk A, Booth GL (2015). Prevalence of Prediabetes and Undiagnosed Diabetes in Canada (2007–2011) According to Fasting Plasma Glucose and HbA&lt;sub&gt;1c&lt;/sub&gt; Screening Criteria. Diabetes Care.

[79] Cowie CC, Rust KF, Byrd-Holt DD, Eberhardt MS, Flegal KM, Engelgau MM (2006). Prevalence of diabetes and impaired fasting glucose in adults in the U.S. Population. Diabetes Care.

